# Physiological Gait versus Gait in VR on Multidirectional Treadmill—Comparative Analysis

**DOI:** 10.3390/medicina55090517

**Published:** 2019-08-22

**Authors:** Katarzyna Jochymczyk-Woźniak, Katarzyna Nowakowska, Jacek Polechoński, Sandra Sładczyk, Robert Michnik

**Affiliations:** 1 Department of Biomechatronics, Faculty of Biomedical Engineering, Silesian University of Technology, Roosevelta 40, 41-800 Zabrze, Poland; 2 Department of Physical Activity and Health Prevention, Faculty of Physical Education, The Jerzy Kukuczka Academy of Physical Education in Katowice, Mikołowska 72A, 40-065 Katowice, Poland; 3 Students Scientific Circle “Biokreatywni”, Faculty of Biomedical Engineering, Silesian University of Technology, Roosevelta 40, 41-800 Zabrze, Poland

**Keywords:** BTS System, energy expenditure, gait analysis, kinematics, OMNI treadmill, virtual reality, physical activity, physiotherapy

## Abstract

*Background and objectives:* Virtual reality (VR) is increasingly often finding applications in physiotherapy and health promotion. Recent years have seen the use of advanced technologies in the promotion of physical activity (PA) in society. New simulators, e.g., treadmills, enable the performance of PA (e.g., locomotive movements) in VR (artificially created virtual world). The question of how such movements are similar to natural forms of human locomotion (march, run) inspired the comparative analysis of physiological gait and gait in VR on a multidirectional Omni treadmill. *Materials and Methods:* The tests involved the use of the BTS Smart system for the triplanar analysis of motion. The test involved 10 healthy females aged 20–24 (weight: 52 ± 3.1 kg, height 162 ± 5.4 cm). Measurements were performed at two stages. The first stage involved the standard assessment of physiological gait, whereas the second was focused on gait forced by the Omni treadmill. The following gait parameters were analyzed: Flexion-extension in the ankle, knee joint and hip joint, rotation in the hip joint and knee joint, foot progression, adduction-abduction in the knee joint and hip joint, pelvic obliquity, pelvic tilt, pelvic rotation as well as energy expenditure and the movement of the body center of mass. *Results:* The analysis of the test results revealed the existence of differences in the kinematics of physical gait and gait on the treadmill. The greatest differences were recorded in relation to the dorsal-plantar flexion in the ankle, the foot progression, the rotation of the knee joint, pelvic tilt and rotation. In addition, the gait on the treadmill is characterized by the longer duration of the stance phase and reduced ranges of the following movements: Flexion-extension in the ankle, knee joint and hip joint, adduction-abduction in the hip joint as well as rotation in the ankle and hip joint. The values of potential, kinetic and total energy recorded in relation to forced gait are significantly lower than those of physiological gait. *Conclusions:* Because of the fact that the parameters of gait on the Omni platform vary significantly from the parameters of physical gait, the application of the Omni treadmill in the re-education of gait during rehabilitation should be treated with considerable care. Nonetheless, the treadmill has adequate potential to become a safe simulator enabling active motion in VR using locomotive movements.

## 1. Introduction

Recent years have seen computer technologies applied in the popularization of physical activity (PA) in society. The aforesaid popularization is, among other things, manifested by the development of the so-called active video games (AVGs), where the player controls the course of the game by movements of their body. In relation to numerous AVGs, the results of the monitoring of physical effort parameters revealed that their values coincided with those recommended for health by international organizations [1,2,3,4,5] and were favorable both for healthy [6,7,8] and unhealthy individuals [9,10,11]. In addition, because of the highly assessed attractiveness of the above-named games, players are capable of extended PA in an interactive form (in comparison with classical one), which can translate into better health [7].

The subsequent stage of applying technological progress to improve public health involves the transfer of AVGs to virtual reality (VR)—a digital space generated by the computer. Users enter the realm of VR wearing special goggles, which separate the players from the real world and make them become part of the game-intensifying emotions accompanying the game [12]. Test results revealed that PA in VR can reduce the perception of pain present during physical exercises, resulting in the reduction of a sense of effort and, consequently, enabling the extension of PA and increasing its attractiveness in comparison with that performed in a classical manner [13]. It was also observed that the training in VR was effective in the rehabilitation of patients suffering from chronic diseases [14,15,16].

The health-promoting nature of PA is largely related to its intensity, which, according to the WHO (World Health Organization), should be at least moderate (<3 METs—the metabolic equivalent of task) [17]. The performance of intensive exercises in VR can be facilitated by appropriate devices. Newly developed simulators enable cycling, walking or jogging in the virtual world. The aforesaid simulators are equipped with sensors reflecting user’s movements in VR. As a result, the user becomes part of the artificially generated world, where the movements of the user’s body make it possible to control the course of a game, sports training or visits to virtual destinations [12,18].

An example of a device enabling the performance of locomotive movements in VR is an Omni treadmill manufactured by the Virtuix company. The above-named equipment interacts with the HTC Vive system and enables the user to safely walk and run in various directions when performing AVGs. The locomotion of the treadmill is specific and results from the treadmill design, differing from other devices of this type. The user moves along a basin-shaped platform wearing slippery footwear.

As a result, the gait on the treadmill seems to differ from physiological gait or run. Gait parameters have been studied for treadmills in VR [19,20,21], but there have not been any studies focusing on a multiodirectional Omni treadmill. In terms of the use of the above-named device in therapy, it is important to assess parameters of locomotive movements using a simulator and to refer the obtained results to normative values. The foregoing is important as regards to potential health-related advantages of possible users of the equipment. This study discusses the comparative analysis of physiological gait and gait on the multidirectional Omni treadmill making it possible to move in VR.

## 2. Materials and Methods

The test group was composed of 10 females aged 20–24 years (weight: 52 ± 3.1 kg, height: 162 ± 5.4 cm). All test participants were healthy and did not report any serious dysfunctions or injuries of their lower limbs in the past. This study was approved by the ethical committee of the Jerzy Kukuczka Academy of Physical Education in Katowice (protocol number 9/2018). Written informed consent was obtained from the patient to publish images.

**Figure 1:**
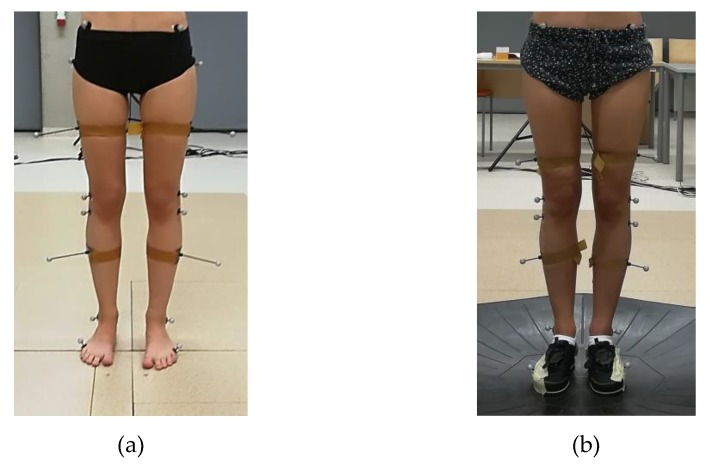
Arrangement of markers on the lower limbs of the person before measurements of parameters of (**a**) physiological gait and (**b**) gait on the Omni treadmill.

**Figure 2:**
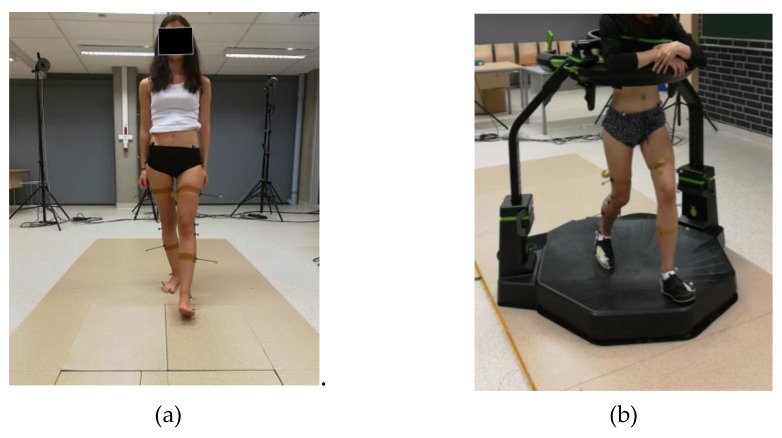
Person tested during measurements of parameters of (**a**) physiological gait and (**b**) gait on the Omni treadmill.

The tests were performed using a system enabling the BTS Smart (BTS S.p.A., Milanese, IT, Italy) triplanar motion analysis. The measurement stand was provided with six IR cameras (sampling rate of 250 Hz) tracking movements of passive markers attached to strictly specified anatomic points of a test participant’s body in accordance with the Davis model [22] (Figure 1a), two video cameras and a computer with a dedicated software programme, i.e., BTS Smart Capture, Tracker and Analyzer, enabling (BTS S.p.A., Milanese, IT, Italy), respectively, the recording of the markers position in space, the definition of the markers in accordance with a previously adapted model and the identification of motion kinematics. The experimental tests were performed at two stages. The first stage involved the standard examination of physiological gait. A person subjected to the test was supposed to walk barefoot, at a natural speed, along a measurement path (Figure 2a). After each passage, the entire gait cycle was selected for analysis. The second stage included tests involving the use of the Omni treadmill (Virtuix Inc, Austin, US-TX, US). The above-named device consists of a slide platform in the form of a basin, extension arms of adjustable height, an openable hoop attached to the extension arms and a harness stabilizing a test participant and preventing them from a fall during measurements. The harness also reads out parameters of a person’s position on the treadmill and uploads them to a computer. When walking on the Omni treadmill, test participants wore special dedicated footwear. The soles of the footwear were provided with elements made of hard plastic, facilitating the loss of grip (slide) when the foot came into contact with the basin surface [23].

The footwear, in the upper area of the foot, was equipped with adapters of trackers (movement sensors), which, during a game, send a signal to a receiver fixed on the treadmill. The receivers send the signal to the computer, analyzing movements of the player’s feet and transferring the movements directly to an activated game or application. A previously prepared test participant, i.e., provided with footwear and markers, put on the treadmill harness and walked on the treadmill at a natural speed (Figure 2b). The first several (between ten and twenty) minutes were not recorded as, during that time, the test participant was to become familiar with the equipment and technique of gait on the treadmill (basin). The recording of kinematics started after 10 min, i.e., when the test participant was able to move on the treadmill without any problems.

From each stage of the tests (of physiological gait and gait forced on the treadmill), ten complete cycles of gait were selected. The following gait parameters were analyzed: flexion-extension in the ankle, knee joint and hip joint, rotation in the knee and hip joint, foot progression, adduction-abduction in the knee joint and hip joint and pelvic obliquity, pelvic tilt, pelvic rotation. The positions of the joints and some movements in the joints (i.e., flexion-extension in the ankle, knee joint and hip joint, rotation in the knee and hip joint, adduction-abduction in the knee joint and hip joint ) were defined in the local coordinate systems of a given segment in accordance with the ISB Coordinate System [24]. The other angles (i.e., foot progression, pelvic tilt, pelvic obliquity, pelvic rotation) are absolute angles, which were measured relative to laboratory axes with the sagittal and transverse axes automatically selected according to the direction of walking. In motion analysis system, foot progression is defined as the angle between the foot vector (projected into the laboratory’s transverse plane) and the sagittal laboratory axis. A positive number corresponds to an internally rotated foot.

The research work also involved analyses of the ranges of pelvis movements in three planes, the range of movements in the joints of the lower limbs, step frequency, energy expenditure and the movement of the center of mass of the body. The value of energy expenditure was identified as the sum of potential energy and kinetic energy of the center of mass normalized in relation to body mass on the basis of a mathematical algorithm discussed in detail in the publication by Michnik et al. [25]. Energy expenditure was determined using a software programme created in the MatLab (The MathWorks, Natick, US-MA, US) environment.

The quantitative variables of analyzed parameters determining energy expenditure and the movement of the center of mass were described using the mean and the standard deviation (SD) based on 10 cycles of gait subjected to analysis. The normality of the distribution of results was verified using the Shapiro-Wilk test. The level of significance adopted in the statistical analyses was *p* < 0.05. The determination of differences concerning analyzed parameters of physiological gait and gait on the Omni treadmill involved the use of (depending on the normality of the distribution of analyzed variables) the Student’s *t*-test or the Wilcoxon test for dependent tests. The calculations were performed using the Statistica software programme, version 12 (Statsoft Inc., Tulsa, US-OK, US).

## 3. Results

**Figure 3:**
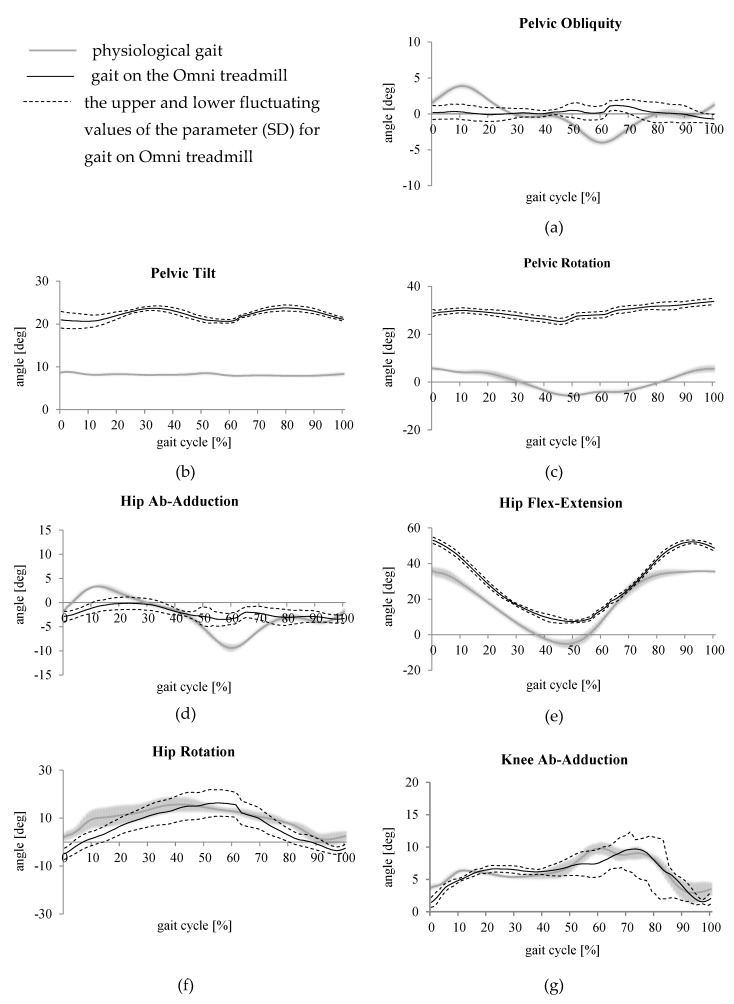

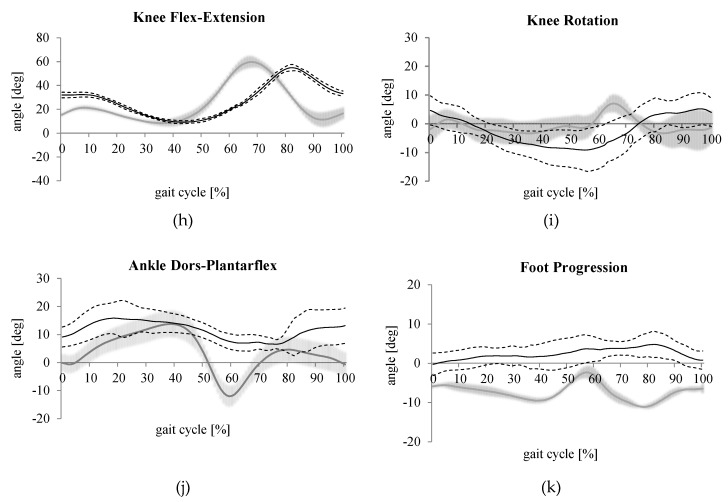
Changes in angular values in relation to the cycles of physiological gait and forced gait on the Omni treadmill in relation to (**a**) pelvic obliquity, (**b**) pelvic tilt, (**c**) pelvic rotation, (**d**) adduction-abduction in the hip joint, (**e**) flexion-extension in the hip joint, (**f**) rotation in the hip joint, (**g**) adduction-abduction in the knee joint, (**h**) flexion-extension in the knee joint, (**i**) rotation in the knee joint, (**j**) dorsal-plantar flexion of the foot, (**k**) foot progression.

**Figure 4:**
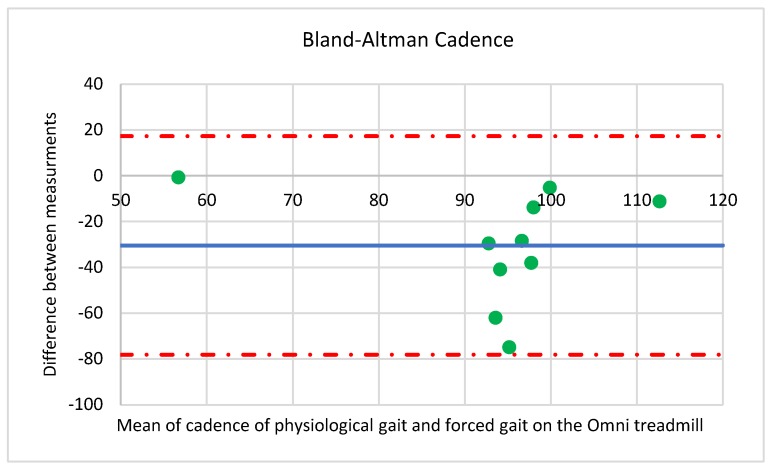
Bland–Altman plot for the step frequency.

Average gait parameters were compared between the Omni treadmill and physiological gait analysis. The grey area in Figure 3 signifies the range of the oscillation of data concerning tests of physiological gait (on the path), the black color denotes the linear courses presenting the upper and lower fluctuating values of the parameter in relation to forced gait, whereas the full line signifies the mean value (Figure 3 and Figure 4).

The analysis of diagrams comparing the kinematics of physiological gait and that of forced gait on the Virtuix Omni treadmill (Figure 3 and Figure 4) revealed the existence of differences in the angular courses in time in the joints of the lower limbs and in the rotation of the pelvis [26]. The range of pelvic obliquity during gait on the treadmill was lower than during physiological gait. The pelvis remained in an unchanged position almost throughout the cycle of gait (Figure 3a). During forced gait, the pelvis was positioned in increased tilt, by approximately 10° in relation to physiological gait (Figure 3b). In addition, during gait on the treadmill basin, the pelvis was positioned in increased internal rotation, by over 20° throughout the cycle of gait (Figure 3c).

Gait on the treadmill was characterized by the reduced range of movements in the hip joint in the frontal plane, the lower limbs were not adducted at the beginning of the gait cycle and abducted at the final part of the stance phase of gait (Figure 4a). In addition, it was possible to observe the excessive flexion of the lower limbs in the hip joint throughout the cycle of gait as well as the lack of clearly visible hyperextension at the final stage of the stance phase (Figure 4b). Moreover, it was possible to notice the increased range of rotating movements in the hip joint during gait on the treadmill basin (Figure 4c). The range of movements in the knee joint in the frontal plane during gait on the treadmill was similar to that during physiological gait (Figure 4d).

Gait on the treadmill was characterized by greater flexion in the knee joint at the beginning of the stance phase, by more than 10°, using the first contact of the foot with the ground. The loading response phase was not accompanied by characteristic flexion in the knee joint. The shift of the diagram indicated the significantly longer duration of the stance phase, i.e., taking the foot off the ground significantly later than in the case of physiological gait (Figure 4e). One of the characteristics of gait on the treadmill was increased rotation in the knee joint (Figure 4f). During gait on the treadmill basin, the foot was positioned in the dorsal flexion. The characteristic plantar flexion of the foot at the end of the stance phase was not observed (Figure 4g). The foot was positioned in increased internal rotation throughout the cycle of gait (Figure 4h). The analysis of the obtained courses of the kinematics of gait on the Omni treadmill indicated the significantly extended duration of the stance phase. Differences in the obtained courses were readily visible. In most cases, the characteristics did not overlap with the ranges of movements being smaller for gait on the treadmill than those in relation to physiological gait. The motion on the Omni treadmill is specific as the tested person is wearing a harness enabling walking in the same place as well as rotation around their axis, the platform is basin-shaped. The design of the treadmill forced test participants to bend forward on the treadmill hoop to be able to walk, which might have affected the obtained results. Gait on the treadmill rather resembled sliding on the surface of the basin.

deg (degree) is a measurement of a plane angle, defined so that a full rotation is 360 degrees The comparative analysis also included step frequency. The gait on the Omni treadmill is characterized by lower step frequency (79.25 ± 24.81 step/min) than physiological gait (109.11 ± 20.90 step/min). The differences in measurements of step frequency during physiological gait and the gait on the Omni treadmill were compared using the Bland-Altman method (Figure 4).

As can be seen in the diagram, the gait on the Omni treadmill is characterized by lower step frequency, on average by 30 step/min with a precision of 24 step/min. Measurement differences were restricted within the range from −78 to 17 step/min.

Table 1 presents the results of kinetic (ΔEk), potential (ΔEp) and total (ΔEc) energy as well as in the movement of the center of mass (COM) in the sagittal plane (ΔSacrum). The table presents the values concerning physiological gait and gait forced on the Omni treadmill, respectively.

## 4. Discussion

Observations of the posture of test participants moving on the Omni treadmill revealed that, when walking in the basin, the participants bent their trunk forwards, resting on the treadmill hoop harness. At times, the participants also placed their upper limbs on the hoop. Gait on the treadmill was characterized by lower step-making frequency.

The data concerning the angular ranges of movements in joints obtained during the tests of natural and forced gait (Table 2) were compared with the values recorded for physiological gait recorded in the publication by Leszczewska et al. [27]. The angular values measured by the authors were similar to those concerning physiological gait obtained in the tests discussed in this study. The greatest deviations were recorded in relation to adduction–abduction and rotation in the knee joint. The angular ranges of movements for individual joints in relation to forced gait on the Omni treadmill significantly varied from the normative values. Forced gait was characterized by the smaller range of flexion-extension and almost two times lower rotation in the ankle. The range of flexion-extension in the knee joint was reduced by approximately 5°. The adduction-abduction and rotation in the knee joint did not vary significantly from the standard. Gait on the basin was characterized by the smaller range of flexion-extension and rotation in the hip joint. In turn, the range of adduction-abduction in the hip joint was almost three times smaller. During gait on the treadmill, the pelvis moved in a significantly greater range in the sagittal plane and in a smaller range in the frontal and transverse planes.

Gait on the basin (“concave” surface) of the Omni treadmill could be compared to uphill walking. The foot was maintained in the dorsal flexion. Franz and Kram [28] performed the comparative analysis of gait inclined at an angle of 9° in relation to the ground (imitation of uphill walking) and physiological gait. The courses of kinematics obtained by the authors significantly overlapped with those of gait forced by the Omni treadmill. In terms of physical activity, the above-named fact should not be treated as unfavorable, nor is it considered detrimental to the locomotor system to walk up the hill if compared to walking on the flat (horizontal) ground. Research performed by Ehlen et al. [29] revealed that, as regards, obese persons, the above-presented form of movement could even be more beneficial than moving on a flat surface. It is recommended that obese persons should perform physical activity characterized by moderate intensity [30], safety (low risk of injuries) and easiness. The above-presented criteria are satisfied by marching recommended to obese persons [31]. However, when walking at a preferred speed, the intensity of physical effort may be overly low to provide physiological benefits [32,33]. As a result, obese persons have to move faster to satisfy health-related recommendations, which in turn, may entail risky consequences as there is a significant positive correlation between the rate of marching and the loading of the lower limbs [34,35]. In their research, Ehlen et al. [29] revealed that slow uphill walking, i.e., at an inclination restricted within the range of 6° to 9°, results in the lower loading of the locomotor system than a fast march on a flat surface without reducing energy expenditure (comparable in both cases). The results of the above-named tests indicate a strategy favorable for obese persons, i.e., maintaining the positive effect of training (reduction of fat tissues) combined with the lower risk of lower limb injuries. Increasing the intensity of physical activity when marching, without additional load applied to joints of the lower limbs, can also be obtained by employing the upper limbs. The aforesaid correlation is visible when comparing physiological gait and nordic walking (NW). It has appeared that NW, performed at the same rate as gait without poles, is characterized by higher energy expenditure [36].

Energy expenditure during gait constitutes important diagnostic information. In worldwide scientific literature, it is possible to come across many publications discussing research dedicated to the determination of energy expenditure in a cycle of gait as well as during the performance of many other activities (e.g., racewalking) [25,37,38,39,40,41,42,43]. The determination of energy expenditure for many different activities demonstrates its value as the source of information. It was revealed that the differences between the values of potential, kinetic and total energy expenditure as well as the movement of COM in relation to physiological gait and gait on the Omni treadmill were statistically relevant. The results obtained in the tests were compared with those found in reference publications. In relation to physiological gait, the average total energy amounted to 0.571 J kg^−1^ m^−1^ and was higher than that obtained in the tests by Chwała et al. [37], where the average value of the total energy in a group of young test participants amounted to 0.36 J kg^−1^ m^−1^. Forced gait on the Omni treadmill was characterised by significantly lower total energy (0.222 J kg^−1^ m^−1^). In addition, locomotion on the Omni treadmill was characterised by, on average, two times lower values of potential energy than physiological march. The foregoing was primarily connected with the lower value of movement indicated by the sacrum marker in the sagittal plane, which, in turn, could be ascribed to the specific nature of walking on the basin, i.e., characterised by a slide component and lower step-making frequency (where in physiological gait the lower frequency at which steps are made is connected with the lower speed of walking).

In terms of the biomechanical assessment of energy expenditure, the use of the Omni treadmill should be subjected to actual energy-expenditure-related tests. Such measurements would require appropriate software programs combined with the treadmill as well as equipment enabling the long-term calorimetric evaluation of performed locomotive activities. Only the assessment of the intensity of physical effort when moving in VR on the Omni treadmill and comparing the value of such effort with recommendations concerning health-promoting physical activity could reveal whether walking or running on the treadmill is sufficiently intensive to state that a regular walk/run is beneficial for health.

Because of the fact that physiological gait varies from locomotive movements performed on the tested treadmill, it is necessary to treat the attempted re-education of gait on the treadmill with adequate care as there is a risk of adopting improper habits. Producers of such equipment should try to create treadmills enabling the performance of locomotive movements as close to natural locomotion as possible.

However, it seems that the Omni treadmill could be used as a simulator enabling safe (protected by the harness in the hoop) and active movements in VR. Because of the slide component of motion and relatively low energy expenditure during gait, the above-mentioned movements in VR could be less demanding (lighter load) for joints of lower limbs than physiological gait. The foregoing is of particular importance for overweight and obese persons. Physical activity in the virtual world is likely to be found attractive by potential users and could increase their motivation for exercising. In addition, the treadmill should intensify the sense of immersion in VR and, consequently, locomotive movements performed during AVGs. However, the aforesaid presumptions should be verified by appropriate tests.

## 5. Conclusions

The use of objective quantitative tools of motion analysis made it possible to define the specific nature of gait on the Omni treadmill. The comparative analysis confronting gait on the treadmill basin with physiological gait revealed the existence of similarities and differences in the kinematics of movements. The greatest deviations were recorded for dorsal-plantar flexion in the ankle, foot progression, knee joint rotation, pelvic tilt, and rotation. The motion on the treadmill was characterized by a significantly longer duration of the stance phase of gait. Forced gait on the Omni treadmill revealed reduced ranges of flexion-extension in the ankle, knee joint and in the hip joint, adduction-abduction in the hip joint as well as rotation in the ankle and knee joint. In turn, the pelvis moved within a greater range in the sagittal plane and a smaller range in the frontal and transverse planes.

The comparison of the test results with data found in related reference publications revealed the similarities of the kinematic variables obtained during the forced gait on the treadmill and uphill walking. However, the values of potential, kinetic and total energy recorded in relation to forced gait were significantly lower than those characteristics of physiological gait.

Due to the fact that the parameters of gait on the Omni treadmill vary from indicators of physiological gait, the use of the above-named device in the re-education of gait during rehabilitation should be treated with extra care. However, the treadmill may potentially become a safe simulator enabling motion in VR using locomotive movements. It can be presumed that the use of the treadmill could intensify the sense of immersion in VR as well as might increase the intensity and attractiveness of AVGs, yet the verification of the aforementioned suppositions requires the performance of appropriate tests.

## Figures and Tables

**Table 1:** Mean values of potential, kinetic and total energy expenditure in relation to each person as well as the movement of center of mass (COM) in relation to physiological gait and gait on the Omni treadmill.

	Physiological Gait Δep [J kg^−1^]	Gait on the Omni Treadmill ΔEp [J kg^−1^]	Physiological Gait Δek [J kg^−1^]	Gait on the Omni Treadmill Δek [J kg^−1^]	Physiological Gait Δec [J kg^−1^]	Gait on the Omni Treadmill Δec [J kg^−1^]	Physiological Gait Δsacrum [M]	Gait on the Omni Treadmill Δsacrum [M]
**Person 1**	0.348	0.202	0.358	0.006	0.551	0.208	0.033	0.007
**Person 2**	0.601	0.244	0.104	0.021	0.704	0.265	0.058	0.021
**Person 3**	0.421	0.154	0.174	0.016	0.595	0.17	0.038	0.012
**Person 4**	0.303	0.12	0.114	0.011	0.416	0.132	0.029	0.008
**Person 5**	0.442	0.244	0.172	0.018	0.614	0.262	0.042	0.018
**Person 6**	0.449	0.081	0.129	0.014	0.578	0.095	0.04	0.005
**Person 7**	0.358	0.177	0.177	0.033	0.535	0.209	0.034	0.013
**Person 8**	0.454	0.315	0.084	0.018	0.538	0.327	0.04	0.025
**Person 9**	0.427	0.251	0.146	0.018	0.573	0.269	0.031	0.02
**Person 10**	0.364	0.284	0.088	0.023	0.452	0.288	0.033	0.024
**Mean**	0.417	0.207	0.155	0.018	0.556	0.222	0.038	0.015
**SD**	0.082	0.074	0.08	0.007	0.081	0.073	0.008	0.007

**Table 2:** Presents the angular ranges of movements in individual joints during physiological gait and gait on the Omni treadmill against normative values. Angular ranges of movements in individual joints during physiological gait and gait on the Omni treadmill against normative values.

	Range of Motion (Min,Max)		
	Normative Values [27]	Physiological Gait	Gait on the Omni Treadmill
Registration System	Vicon	BTS Smart	BTS Smart
Age [years]	18–40	20–24	20–24
Ankle dorsal-plantar flexion [deg]	25.5	25.8 (−12,13.7)	9.3 (6.6,15.9)
Foot progression [deg]	15.7	13.5 (−11.1,−2.3)	5.1 (−0.3,4.8)
Knee flexion-extension [deg]	56.7	51.3 (8.4,59.7)	45.7 (9.2,54.9)
Knee adduction-abduction [deg]	13.4	6.9 (2.9,9.8)	8.7 (1.4,9.7)
Knee rotation [deg]	16	10.4 (−3.4,7.1)	14.4 (−9.2,5.2)
Hip flexion-extension [deg]	43.3	41 (−5.2,35.8)	45. 6 (7.4,53)
Hip adduction-abduction [deg]	11.6	12.8 (−9.4,3.4)	3.4 (−3.5,−0.1)
Hip rotation [deg]	13	14.7 (1,15.7)	21.3 (−4.9,16.4)
Pelvic tilt [deg]	2.8	0.9 (7.9,8.8)	3.1 (20.6,23.8)
Pelvic obliquity [deg]	8.4	7.9 (−4,3.9)	1.9 (−0.7,1.2)
Pelvic rotation [deg]	9.2	11.3 (−5.7,5.7)	8.5 (1,1.7)
